# Materials and Processes for Sustainable Energy and Environmental Systems

**DOI:** 10.3390/ma15196692

**Published:** 2022-09-27

**Authors:** Natalia Howaniec

**Affiliations:** Department of Energy Saving and Air Protection, Central Mining Institute, Pl. Gwarków 1, 40-166 Katowice, Poland; n.howaniec@gig.eu

Developments in materials and processes for sustainable energy and environmental systems are of special importance in the world of depleting natural resources, serious environmental concerns, increasing energy demand, and disturbances in the global energy market affecting energy supply chains. Numerous advancements have been made so far in various sectors of the economy in terms of low-emission energy-system development, including gasification to synthesis gas and hydrogen-rich gas, with greenhouse gas emission mitigation [1,2,3,4], energy efficiency improvements, and waste valorization [5,6,7,8]. This Special Issue intends to present recent developments in various aspects related to materials and processes contributing to the creation of sustainable power systems and environmental solutions. These include, in particularly, materials and engineering processes for low-emission energy systems, carbon capture and utilization technologies, hydrogen economy, functional carbon materials, advanced energy materials, as well as waste and byproduct valorization in energy systems and environmental engineering.

The basics of coal-derived fluidized bed fly ash valorization in the process based on a direct semi-dry carbonation method with the use of 60%vol. carbon dioxide in air at a temperature range of 40–100 °C was tested at a laboratory scale in a thermogravimetric analyzer [9]. A dynamic model describing the carbonation process was also developed, and fitting of the experimental data to this model was performed with the use of the Levenberg–Marquardt algorithm. This work contributed to previously reported studies on the utilization of carbonated fly ash from conventional and fluidized bed boilers in the production of cementitious materials. Carbon dioxide mineralization with the use of various waste streams as sources of alkaline earth metals and the direct and indirect carbonation methods in wet, semi-dry, or dry conditions is widely discussed in the literature and considered as a potential element of carbon capture and utilization technologies and industrial waste valorization. Still, the economics of the up-scaled process may be discouraging without relevant financial and policy incentives and/or cutting-edge advancements in otherwise cost- and energy-intensive technological schemes.

Self-heating and spontaneous combustion phenomena cause safety and environmental concerns in energy resource extraction, handling, and post-process residues management. The experimental study on the emission of selected gases in the process of self-heating of coal, simulated in a flow reactor in a temperature range of 50–250 °C proved carbon dioxide to be the main oxidation product [10]. The hierarchical clustering analysis applied to the experimental data showed that coal samples with higher oxygen, sulfur, and inertinite contents and lower ash and carbon contents released larger total volumes of gases in the self-heating process than the remaining coal samples tested. It was also reported that the carbon dioxide to carbon monoxide volume ratio decreased with an increase in the process’s temperature and that the effects of self-heating of coals of similar ranks might differ because of differences in their physical and chemical properties.

Industrial processes result in the generation of significant volumes of heavily contaminated wastewater and sludge that need to be treated with the application of various chemical, physical, or biochemical methods before reuse or landfilling. The experimental study on the treatment of chemically complex wastewater from the tannery industry was performed with the use of an effective reagent of a combined oxidation and coagulation effect and low negative impact on the environment, i.e., potassium ferrate [11]. The robust statistical design Taguchi method (TM) and the response surface methodology (RSM) based on central composite designs (CCDs) were employed in an experimental study for process optimization. While the first method enabled the selection of the most favorable combination and values of input parameters, the second allowed for a more precise statistical analysis of the experimental data acquired. Potassium ferrate was proven to efficiently reduce color, chemical oxygen demands, and the total organic carbon of the wastewater treated. The RSM method was shown to be a flexible method for planning and the optimization of wastewater treatment technologies, while the TM method was reported to be useful in the preliminary stage of the process’s optimization, since it requires the performance of a lower number of experiments and simplifies the statistical analysis of the data.

A wide reuse of waste materials lies at the basis of a circular economy. Municipal wastewater treatment plants produce considerable amounts of sewage sludge that may be utilized for energy purposes instead of landfilling. The economic feasibility of the method of sewage-sludge stabilization and reuse as a potential renewable energy source was assessed on the basis of the experimental results and operational performance of a pilot-scale installation for sewage-sludge homogenization, bioconversion, molding, and thermal stabilization, followed by the utilization of the final process product in co-combustion processes [12]. The results showed that the municipal sewage sludge processed in the proposed technological scheme may constitute an alternative biofuel, entailing no additional unit costs of electricity generation when replacing a part of fossil fuels in combustion systems.

Solvents are widely applied in industrial-scale processes, e.g., in an organic synthesis. A five-membered heterocyclic organosulfur sulfone (R-SO_2_-R’, where R/R’ is an alkyl, alkenyl, or aryl) is commonly applied as an extractive solvent in liquid–liquid and liquid–vapor extraction processes. Under elevated temperatures and in the presence of oxygen, water, or chlorides, it may decompose with a release of SO_2_ and the formation of a corrosive H_2_SO_3_. A pilot case study on a corrosion process in low conductivity sulfolane-based fluids with the use of an industrial multi-electrochemical technique was performed to evaluate the effect of an aqueous phase on a general and a localized corrosion of AISI 1010 carbon steel [13]. The extent of corrosion was evaluated with the use of an open-circuit potential method, potentiodynamic polarization curves, scanning electron microscopy with an energy dispersive spectroscopy system (SEM/EDS) and scanning Kelvin probe techniques. An increase in water content (1–3%vol.) in sulfolane-based fluids was proven to deteriorate the resistance of the steel tested to uniform and pitting corrosion, as a result of the higher conductance of the fluids.

The progress in rational and efficient resource use as well as in the development of advanced functional materials for energy and environmental applications is evident. Nevertheless, a more interdisciplinary approach in power, material, chemical, and environmental engineering is still needed to build up synergetic effects for a sustainable future.

## References

[1] Krawczyk P., Howaniec N., Smoliński A. (2016). Economic efficiency analysis of substitute natural gas (SNG) production in steam gasification of coal with the utilization of HTR excess heat. Energy.

[2] Smoliński A., Howaniec N., Bąk A. (2018). Utilization of energy crops and sewage sludge in the process of co-gasification for sustainable hydrogen production. Energies.

[3] Smoliński A., Howaniec N. (2017). Chemometric modelling of experimental data on co-gasification of bituminous coal and biomass to hydrogen-rich gas. Waste Biomass Valori..

[4] Smoliński A., Howaniec N. (2020). Hydrogen energy, electrolyzers and fuel cells—the future of modern energy sector. Int. J. Hydrogen Energy.

[5] Zdeb J., Howaniec N., Smoliński A. (2019). Utilization of carbon dioxide in coal gasificationAn experimental study. Energies.

[6] Howaniec N. (2019). Combined effect of pressure and carbon dioxide activation on porous structure of lignite chars. Materials.

[7] Howaniec N., Smoliński A. (2018). Porous structure of Andropogon Gerardi derived carbon materials. Materials.

[8] Howaniec N. (2019). Olive pomace derived carbon materials—Effect of carbonization pressure under CO_2_ atmosphere. Materials.

[9] Łączny M.J., Iwaszenko S., Smoliński A. (2021). Process kinetics of the carbonation of fly ashes: A research study. Materials.

[10] Wojtacha-Rychter K., Smoliński A. (2020). Profile of CO_2_, CO, and H_2_ emissions from thermal oxidation of Polish coals. Materials.

[11] Kozik V., Barbusiński K., Thomas M., Środa A., Jampilek J., Sochanik A., Smoliński A., Bąk A. (2019). Taguchi method and response surface methodology in the treatment of highly contaminated tannery wastewater using commercial potassium ferrate. Materials.

[12] Smoliński A., Karwot J., Bondaruk J., Bąk A. (2019). The bioconversion of sewage sludge to bio-fuel: The environmental and economic benefits. Materials.

[13] Bąk A., Losiewicz B., Kozik V., Kubisztal V., Dybal P., Świetlicka A., Barbusiński K., Kus S., Howaniec N., Jampilek J. (2019). Real-time corrosion monitoring of AISI 1010 carbon steel with metal surface mapping in sulfolane. Materials.

